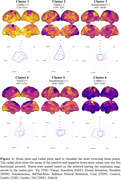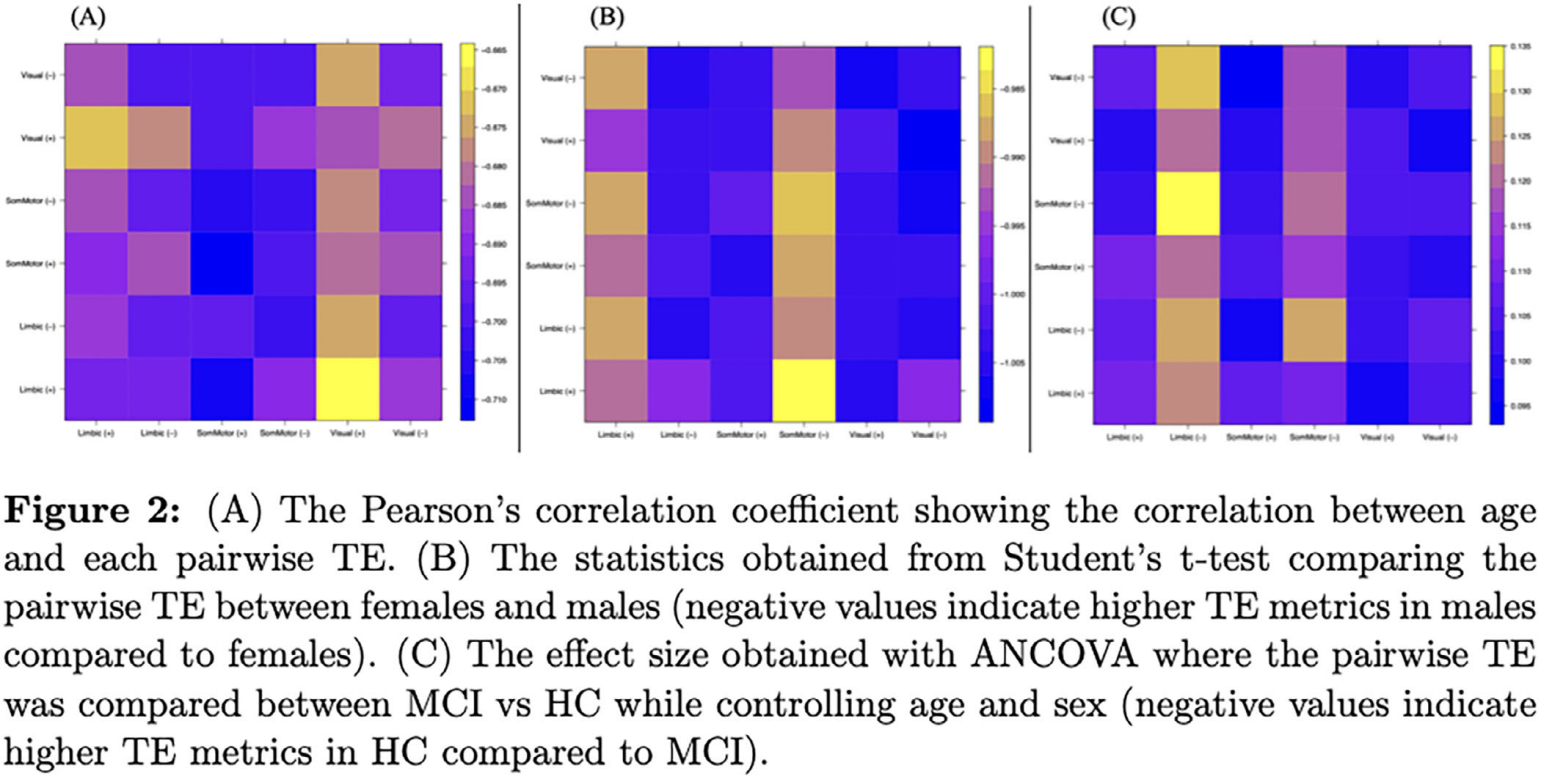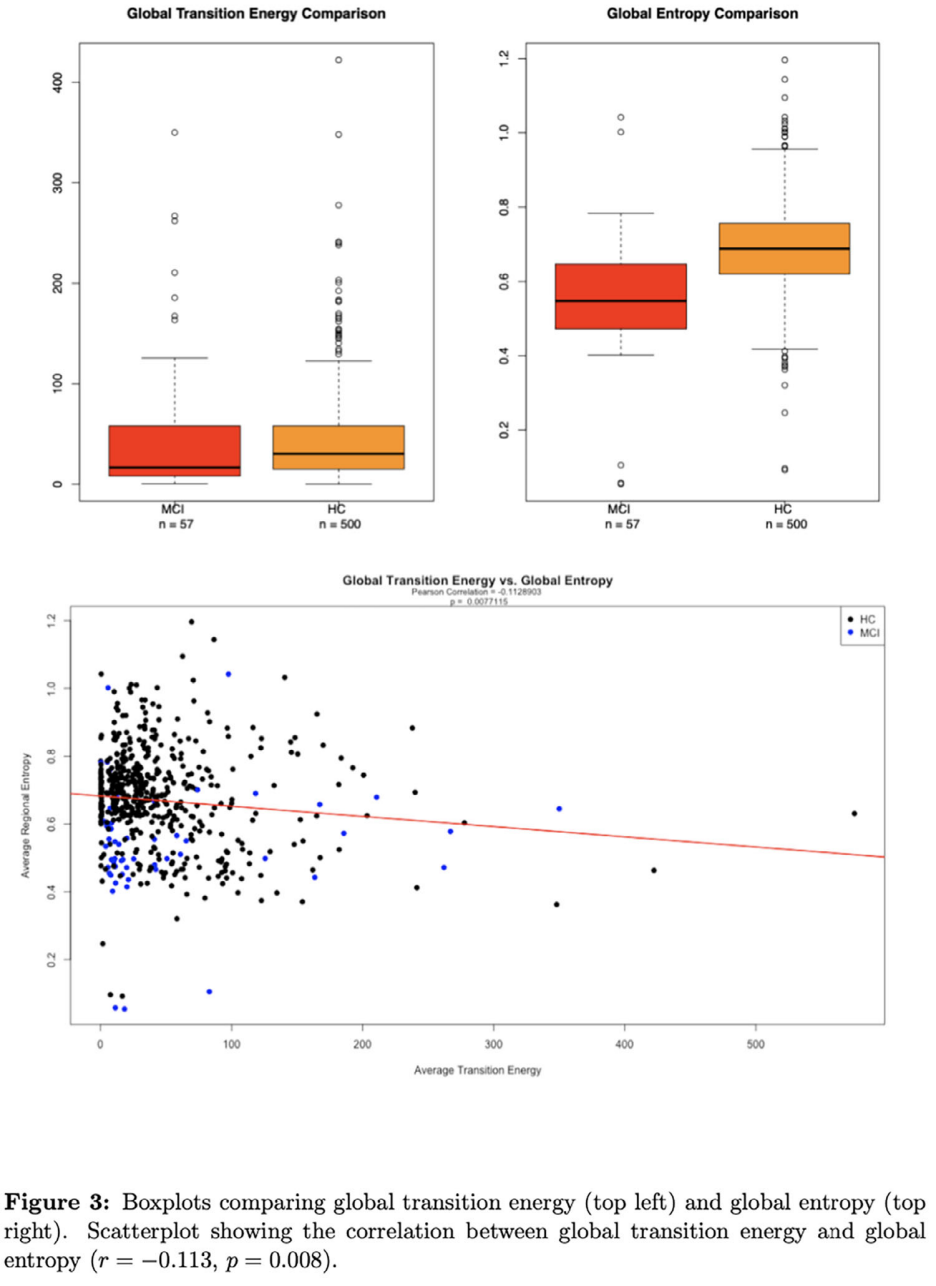# Decreased entropy of functional MRI signals in individuals with mild cognitive impairment compared to cognitively normal controls

**DOI:** 10.1002/alz.087627

**Published:** 2025-01-09

**Authors:** Dara L Neumann, Ceren Tozlu, Qolamreza R Razlighi, José A. Luchsinger, Yaakov Stern, Davangere Devanand, Amy Kuceyeski

**Affiliations:** ^1^ Cornell University, Ithaca, NY USA; ^2^ Weill Cornell Medicine, New York, NY USA; ^3^ Columbia University Irving Medical Center, New York, NY USA

## Abstract

**Background:**

Mild cognitive impairment (MCI) is a clinical cognitive deficit that is not severe enough to meet the threshold for Alzheimer's Disease (AD); however, MCI patients have an increased risk of developing AD. Therefore, a diagnosis of MCI may represent a critical turning point in the trajectory of developing AD. Establishing neurological signatures of MCI using network control theory (NCT) may allow more informed diagnosis, and an understanding of its underlying mechanisms could pave the way for novel treatments.

**Method:**

Functional MRI (fMRI) metrics were collected in MCI patients (n = 57, mean age = 66.68) and healthy controls (HC) (n = 500, mean age = 72.25). The average structural connectivity matrix was obtained using age‐matched controls from the Human Connectome Project‐Aging dataset. Commonly recurring brain states were identified via k‐means clustering of activation matrices over 200 regions using the Schafer atlas. NCT was used to compute the transition energy (TE): the minimum energy required to transition between each pair of brain states. The entropy of each region’s activity was calculated using SampEn, and was then correlated with TE using Pearson’s correlation. Pairwise/global (average of all brain state pairs) TE and global entropy (average of all regions) were compared between MCI and HC using ANCOVA with age and sex as covariates.

**Result:**

The brain states identified via k‐means clustering were high and low amplitude activity in the visual, somatomotor, and limbic networks (Figure 1). While there were no significant differences in pairwise or global TE between MCI and HC (Figures 2‐3), MCI had significantly lower global entropy than HC. ANCOVA revealed that increased age is associated with increased entropy. Pearson correlation showed a significant inverse relationship between global TE and entropy across individuals (r = −0.13) (Figure 3).

**Conclusion:**

Entropy of brain activity measured with fMRI is a promising neuroimaging biomarker of MCI, which is often underdiagnosed or diagnosed with delay. Future work will investigate regional entropy reduction patterns between MCI and AD patients to establish the use of these metrics in disease progression, and to get a more detailed picture of brain activity changes in individuals with these diagnoses.